# Positive Psychological Interventions for Patients with Type 2 Diabetes: Rationale, Theoretical Model, and Intervention Development

**DOI:** 10.1155/2015/428349

**Published:** 2015-04-29

**Authors:** Jeff C. Huffman, Christina M. DuBois, Rachel A. Millstein, Christopher M. Celano, Deborah Wexler

**Affiliations:** ^1^Department of Psychiatry, Massachusetts General Hospital, Boston, MA 02114, USA; ^2^Harvard Medical School, Boston, MA 02115, USA; ^3^MGH Diabetes Center, Department of Medicine, Massachusetts General Hospital, Boston, MA 02114, USA

## Abstract

Most patients with type 2 diabetes (T2D) have suboptimal adherence to recommended diet, physical activity, and/or medication. Current approaches to improve health behaviors in T2D have been variably effective, and successful interventions are often complex and intensive. It is therefore vital to develop interventions that are simple, well-accepted, and applicable to a wide range of patients who suffer from T2D. One approach may be to boost positive psychological states, such as positive affect or optimism, as these constructs have been prospectively and independently linked to improvements in health behaviors. Positive psychology (PP) interventions, which utilize systematic exercises to increase optimism, well-being, and positive affect, consistently increase positive states and are easily delivered to patients with chronic illnesses. However, to our knowledge, PP interventions have not been formally tested in T2D. In this paper, we review a theoretical model for the use of PP interventions to target health behaviors in T2D, describe the structure and content of a PP intervention for T2D patients, and describe baseline data from a single-arm proof-of-concept (*N* = 15) intervention study in T2D patients with or without depression. We also discuss how PP interventions could be combined with motivational interviewing (MI) interventions to provide a blended psychological-behavioral approach.

## 1. Introduction

*Health Behavior Adherence in Type 2 Diabetes*. Approximately 40% of premature deaths in the United States are preventable and related to health behaviors such as diet and physical activity [1, 2], making it critical to identify effective health behavior interventions. This is especially true for patients with existing medical illness, for whom poor health behavior adherence can greatly increase morbidity and mortality [3, 4]. Indeed, across chronic medical conditions, the World Health Organization has stated “increasing the effectiveness of adherence interventions may have a far greater impact on the health of the population than any improvement in specific medical treatments” [5].

Health behavior adherence may be particularly important for patients with type 2 diabetes (T2D), a condition that affects 12% of American adults and is the 7th leading cause of death in the US [6]. Evidence-based care for T2D can lead to good prognosis and high quality of life, but most T2D patients struggle to adhere to one or more major cornerstones of self-management: physical activity, healthy eating, and medication use [7–9]. Indeed, a third of T2D patients have suboptimal medication adherence [10], over half have an “unhealthy” diet [8], and two-thirds have little physical activity [9]. Nonadherence in T2D is independently associated with poor glycemic control, increased rates of vascular, renal, and other complications, and mortality [7, 8, 11].

Current approaches to improve health behaviors in T2D and other illnesses have demonstrated variable effectiveness [12], and successful interventions are often complex and intensive [13–15], making practical implementation a challenge [16]. Hence it is vital to develop interventions that are simple, well-accepted, and applicable to a wide range of patients who suffer from T2D.

*Psychological States, Health Outcomes, and Health Behaviors*. One important and potentially modifiable pathway to improving behavioral adherence in T2D is through psychological interventions. Psychological states may significantly impact health behavior and clinical outcomes in patients with T2D [17]. Negative psychological syndromes such as depression and anxiety have been consistently associated with poor outcomes in patients with T2D [18–20]. For example, depression is associated with impaired glucose control [21], functional disability [22], end-organ complications [21], and mortality [21, 23, 24].

In contrast to negative syndromes, positive psychological states may play a substantial and independent beneficial role in health outcomes. A meta-analysis of 26 studies (*N* > 4000) found that psychological well-being was associated with lower rates of mortality in both initially healthy and medically ill populations, independent of traditional risk factors [25]. In T2D, positive psychological states (e.g., positive affect, well-being) are prospectively associated with lower HbA1c, fewer diabetes complications, and lower rates of cardiac events/mortality [26–28], and Tsenkova et al. [29] found that positive affect at baseline was prospectively associated with reduced HbA1c at 2-year follow-up in older women, independent of multiple covariates.

These beneficial effects of positive psychological states may be mediated through health behaviors. Studies have consistently found links between increased positive psychological states and greater participation in health behaviors in medical populations. For example, Shepperd and colleagues [30] found that higher levels of optimism measured at the outset of cardiac rehabilitation predicted greater improvements in physical activity and saturated fat intake. A pair of analyses found optimism to be associated with greater improvement in dietary quality, controlling for baseline diet, over one year in postmenopausal women [31, 32]. In an observational study of patients with recent acute coronary syndrome, participants with greater optimism 2 weeks after event had greater physical activity at 6 months, controlling for baseline physical activity and overall baseline health status [33]. Each of these studies has been prospective rather than correlational, and each controlled for medical covariates and baseline health behaviors (suggesting that it is not simply that being healthier or more active led to having more positive emotions).

Importantly, positive psychological states are not simply the flipside of depression. Constructs like optimism (*r* = 0.3) and gratitude (*r* = 0.2–0.3) are only somewhat correlated with depression [34, 35], suggesting that nondepressed persons with T2D may still have substantial deficits in positive states. Furthermore, the effects of positive states on health behaviors and outcomes in medical illnesses persist even after accounting for negative affect/depression [25, 28, 29, 36].

Given the empirical evidence identifying specific and prospective links between positive psychological states and health behaviors/outcomes and the multiple potential avenues (e.g., motivation, self-regulation, and concentration [37–39]) by which positive states may lead to greater participation in health behaviors, cultivating these states may have a meaningful impact on completing vital self-care in T2D. This in turn could improve glycemic control and prognosis in this common and disabling condition.

*Aims of This Report*. This paper has four primary goals. First, we will describe the rationale for a positive psychological (PP) intervention in patients with T2D and outline a potential model by which PP interventions may lead to greater participation in health behaviors. Second, we will describe a PP intervention developed for patients with T2D, with specific details about the PP exercises used in this program. Third, we will briefly describe recruitment and baseline data for participants in a small proof-of-concept trial of PP for T2D. Finally, we will discuss motivational interviewing (MI) and related health behavior interventions and how PP might be combined with these interventions to improve outcomes.

## 2. Methods

### 2.1. PP Interventions

PP interventions may represent a promising strategy to increase positive psychological cognitions and emotions in patients with T2D and other chronic conditions. PP is a branch of clinical and research interventions that uses systematic exercises (e.g., gratitude letters, acts of kindness) to target positive cognitive and emotional states [40]. In contrast to complex multicomponent health behavior or psychology interventions, PP exercises are simple, can be delivered remotely (e.g., by phone), and require minimal training of providers [34, 41, 42].

PP interventions have been used in over 4000 mostly healthy subjects and have been found to increase well-being and decrease depression [41, 43], with effect sizes comparable to other psychological interventions [44, 45]. More recently, randomized controlled trials of PP-related interventions in heart and lung disease patients led to improvements from baseline health behaviors (medication adherence and physical activity) [46–48]. Our team has gained substantial experience delivering PP interventions in patients with medical and psychiatric illness [42, 49, 50], and we also recently found that PP interventions led to improvements in self-reported health behavior (diet, exercise, and medication adherence) in a cohort of patients with cardiovascular disease [51].

As opposed to standard, disorder-based psychological interventions, a PP intervention can be delivered to the vast majority of patients who have T2D, rather than applying only to the subset of T2D patients with depression, anxiety disorders, or other clinical psychiatric conditions [52]. Furthermore, there is often substantial variability among patients with medical illness with regard to education, health literacy, motivation, time constraints, and functional status. PP-based exercises do not require high educational attainment or significant time commitment, and they have been enjoyable for patients (and completed at high rates) in diverse medical cohorts [42, 53].

To our knowledge, PP interventions have never been tested in a T2D population, outside of a single small positive affect skills intervention trial that led to improvements in depressive symptoms [54]. If PP is feasible and can similarly boost affect and motivation in patients with T2D who are nonadherent to key health behaviors, it could become a cost-effective tool in the armamentarium to improve functional and medical outcomes in this chronic condition.

### 2.2. Theoretical Model (Figure 1)

**Figure 1:**
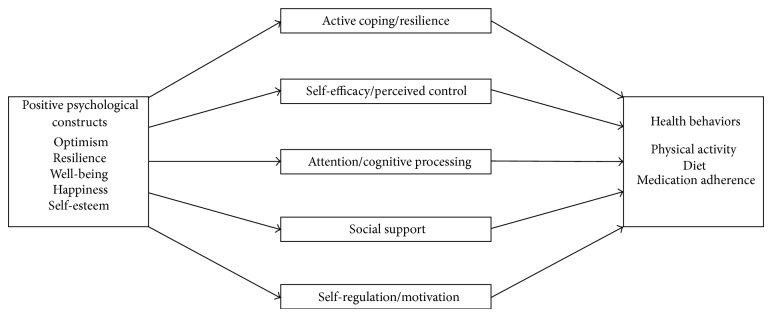
Conceptual model outlining the proposed mediators between positive affect and improved health behaviors.* Note*. This model displays relationships in one direction, though there are bidirectional relationships between most constructs (e.g., being more physically active leads to positive affect).

How might boosting positive affect, optimism, and psychological well-being lead to better health behavior adherence? We present a conceptual model of proposed mediation pathways from which additional empirical support is generated (Figure 1; also see Figure 3 for integration of PP and motivational interviewing interventions, discussed in Section 4.4). In this discussion, we will focus on evidence suggesting that positive psychological well-being leads to improvement in mediational factors linked to health behaviors. However, the relationships within this model are clearly bidirectional; for example, it is certainly true that physical activity can lead to positive emotions, and healthy eating can lead to greater energy.

Broadly, positive states are thought to improve people's social, psychological, and physical resources [55–57]. Positive affect is also linked to improved coping and problem-solving skills [56, 58]. Using this framework and the existing literature, we have identified five potential mediators. Positive psychological constructs appear to affect* attention and cognitive processing*. Positive experiences and cognitions can help people better attend to and process health information [37], including unbiased and open appraisal of negative health information [56]. Self-monitoring is a critical health management task that requires careful attention to health stimuli and leads to improved diet and physical activity [59] along with greater medication adherence [60]. Such processing also results in more accurate perceptions of risk that can lead to health behavior change [37].

Related constructs,* self-regulation and motivation*, also likely play a mediating role. Positive affect can restore or improve self-regulation [38] and goal-directed behaviors [61]. This may be particularly true in the context of perceived threats to health [37]. Positive states are also associated with increased self-control and motivation toward specific goals [62]. Improved self-control and motivation are key drivers in meeting T2D-relevant health behavior goals [39, 63].

*Active coping and resilience* appear to be another main mechanism by which positive affect leads to health behaviors. As described in Pressman and Cohen [64], positive affect may have stress-buffering effects that allow for a broader range of adaptive and creative coping skills [56], and active coping has been shown to improve diabetes self-management [58, 65, 66]. Positive coping shares roots with resilience, which has also been shown to impact health behaviors such as diet, physical activity, and overall T2D self-management [67–69].

*Self-efficacy and perceived control* are influenced by positive cognitions and emotions and are associated with improved health behaviors [17, 58]. A large literature supports these effects [69–71], and specific self-efficacy interventions have been utilized to target health behaviors [72–75], though such interventions may not affect the other mediators of health behavior in the present model.

Positive psychological states and attributes can also increase* social interactions *and* perceived social support*. Optimism and positive affect appear to lead to greater social outreach and perception of social support [76], and people with higher ratings of positive psychological well-being report more social satisfaction [64, 76]. Social support, in turn, is linked with uptake and maintenance of health behaviors, including diabetes self-management [58, 77, 78].

Aside from these five domains, other factors may also play a role in connections between psychological well-being and health. For example, positive states may improve energy and vitality [64, 79], which can increase health behaviors and lead to better health [80].

### 2.3. PP Intervention Design and Development

Utilizing our experience with PP interventions in other populations, we created a PP intervention for patients with T2D that was designed to be tested in an initial proof-of-concept trial.

#### 2.3.1. Intervention Outline (Box 1)

The 12-week intervention consists of 7 distinct PP exercises, to be completed weekly for the first 4 weeks and then biweekly over the next 8 weeks.

*Initial Session (Week 1)*. During the initial enrollment visit in the hospital or clinic, participants receive a treatment manual. At that time, the study interventionist/trainer reviews the introductory portion of the manual. The interventionist describes the overall goals of the intervention, to complete specific positive psychology exercises that boost positive thoughts and feelings and to increase one's focus on positive psychological states in daily life by translating the exercises into skills that can be used regularly. He or she then details specific strategies for deriving optimal benefit from the exercises.

To optimize interaction and engagement at the outset of the intervention, the interventionist may complete the first exercise (Gratitude for Positive Events) together with the participant (in person at enrollment or by phone at the first phone session), with the interventionist soliciting responses and encouraging elaboration by the participant. The participant can also choose to complete the first exercise independently with review of the exercise by phone. In either case, after discussion of the initial exercise, the subsequent week's exercise is outlined and assigned, and a phone appointment is made for one week later.

*Phone Sessions (Weeks 2–12)*. In subsequent weeks, exercises are completed independently by participants and recorded in their treatment manual. Calls (15–30 minutes) with the study interventionist are then carried out weekly or biweekly, correlating with the exercise assignment schedule (i.e., weekly for the first 4 weeks and biweekly thereafter). Phone sessions include review of the prior exercise, consideration of how the exercise skills can be adapted to daily life, and assignment of the next exercise via guided review of the PP manual. In the final week, after exercise review, the interventionist and participant discuss future implementation and maintenance of the exercises/principles in day-to-day life. They also complete together a grid that plans positive psychological activities to be completed over the next 4 weeks; these could be repetition of previous exercises or smaller, more frequent adaptations of the exercises.

Key themes discussed by interventionists across the weeks include (1) developing a greater and more nuanced vocabulary for describing positive thoughts and feelings, (2) learning to savor and “bookmark” positive feelings and experiences, (3) learning redirection toward positive thoughts/feelings if becoming distracted or dysphoric during an exercise, and (4) utilizing novelty in completing the exercises—the idea that performing tasks that they may not have normally completed in their daily lives can lead to even larger increases in positive affect.

#### 2.3.2. Rationale for Phone-Based Intervention

We chose to develop a remotely delivered intervention to address the issue that patients with T2D may have significant functional limitations, transportation challenges, or insufficient time to attend in-person visits [81–83]. Although internet-based interventions can also be useful, a phone-based intervention provides a more personal experience. Our qualitative interviews with medically ill patients have found that such patients consistently express a preference for phone over internet because of this personal connection [84]. In addition, a meaningful subset of T2D patients will not have ready internet access (especially those of lower SES and underrepresented minorities, two groups known to have lesser internet access [85, 86]); thus, a phone-based solution can be accessed by a greater number of individuals.

#### 2.3.3. Intervention Manual

The PP treatment manual (see Boxes 2 and 3 for sample pages; full intervention manual available from the authors) was similar to our manuals designed for patients with depression [50] and heart disease [42], with modest adjustments for patients with T2D. These changes included modified descriptions in the manual regarding the intervention rationale (to reflect T2D) and an extended duration of the intervention (and manual) to 12 weeks, to prepare for future studies that will assess changes in glycemic control over a 12-week period.

The manual contains a general introduction section that describes PP, outlines the use of systematic exercises to improve mood, optimism, and self-confidence, and describes potential connections between positive psychological states and improved self-management of T2D. The main portion of the manual contains sections representing the seven weekly exercises. Each exercise has a short introduction describing the rationale for the exercise and a formal set of instructions. Following the instruction pages, the manual contains space to record the exercise and its effects along with an area to provide feedback about the ease and utility of the exercise.

The final section of the manual contains two sections. First, the manual utilizes a grid to allow participants to schedule specific positive activities in the four weeks following the exercise to allow continuation of activities after intervention and encourage habitual use of the exercises. Second, the final pages of the manual are devoted to a “Favorite Skills” section in which activities that are particularly salient or useful can be recorded for easy access and use.

#### 2.3.4. Training Procedures and Interventionist Adherence

The study interventionist for the subsequent proof-of-concept trial (CD) was trained in several stages by staff (JH, CC) experienced in delivering PP interventions. First, she completed a guided review of the project's provider manual that contains additional information on the rationale and procedures for each exercise, provides guidance for maintaining the focus of the interaction solely on the PP exercise and its review, and conveys specific advice to convey to participants to facilitate completion of the given exercise (e.g., methods of brainstorming).

The interventionist then viewed videos of sample PP sessions, completed PP background reading (book chapters and papers), and personally completed all PP exercises to be used in the intervention. Finally, she completed observed role plays with feedback to gain experience performing and reviewing each exercise. For quality control, sessions were reviewed by a multidisciplinary study team at weekly meetings, and specific feedback about intervention delivery and fidelity was provided in an ongoing manner.

### 2.4. Exercises (See Box 3 for Sample and Box 1 and Table 1 for Structure)

The PP exercises were selected based on their use in prior studies and their superior performance in our prior PP intervention studies. These included PP exercises tested in a broad variety of populations [41, 87] and patients with medical illness [53] and also included exercises from our team's own work providing PP interventions to patients with psychiatric conditions [50] and medical illness [42, 45].

*Gratitude for Positive Events [88] (Week 1)*. Participants recall three events, small or large, in the preceding week that were associated with satisfaction, happiness, pride, or other positive states.

*Using Personal Strengths [40] (Week 2)*. Participants undergo a brief assessment of personal strengths and then find a new way to use one identified strength in the next 7 days.

*Gratitude Letter [40] (Week 3)*. Participants write a letter of gratitude thanking a person for an act of kindness; participants can, at their discretion, share the letter with the other person.

*Enjoyable and Meaningful Activities [89] (Week 4)*. Participants complete a series of self-selected activities that vary between those that bring immediate boosts in mood and those that are more deeply meaningful.

*Recalling Past Success [45] (Week 6)*. Participants recall a prior event in which they experienced success. They write about the event, the positive feelings evoked, their own contribution to the success, and the feelings generated by recalling the event.

*Participant's Choice (Weeks 8 and 10)*

*(i) Repeating an Exercise*. Given evidence that being allowed choice of PP exercises enhances the impact of the intervention [90], and our desire to allow participants some individualization of the intervention, participants select the exercise(s) (from the prior weeks' exercises) they would like to repeat. The study trainer and participant discuss the exercise and any slight alterations.

*(ii) Performing Acts of Kindness [91]*. If participants prefer to complete a new exercise, they can also be assigned the acts of kindness exercise. Participants are instructed to complete three acts of kindness in one day. The acts can be small or large, planned or spontaneous, but must be performed expressly to be kind to another.

Week 12 is used to review the prior (Week 10) exercise and to create the plan/schedule for additional positive interventions over the following four weeks.

### 2.5. Feasibility Study—Design and Baseline Data

The intervention was then tested in a 12-week single-arm proof-of-concept study.

#### 2.5.1. Overview

The goal of this small initial trial was to test logistics, show feasibility and acceptability, and adapt the intervention for future studies based on study outcomes and participant feedback. Institutional review board approval from our healthcare system was obtained prior to any study procedures. The study has completed enrollment, and study procedures and follow-up are ongoing.

#### 2.5.2. Study Criteria

English-speaking adult patients who had (1)* T2D* (meeting ADA criteria [92] for T2D (e.g., HbA1c >6.5%, fasting glucose >126 mg/dL), with diagnosis confirmed by the participant's medical clinician) and (2)* suboptimal adherence* (score <15/18 on Medical Outcomes Study Specific Adherence Scale (MOS SAS); [93] items for diet, exercise, and medications) were potentially eligible for the trial. We selected this MOS SAS cutoff because it required at least mild nonadherence but allowed inclusion of the majority of T2D patients to ensure a broadly applicable intervention.

Exclusion criteria were (1) cognitive impairment precluding consent or meaningful participation in the PP exercises, assessed using a six-item screen developed for research [94], and (2) lack of access to telephone (given that the intervention was delivered by phone).

#### 2.5.3. Recruitment and Baseline Assessments (Table 1)

Participants were enrolled from the outpatient diabetes center and inpatient medical units of an urban academic medical center; we included both populations to capture both stable outpatients and those with more significant comorbid medical illness. In both locations, research staff introduced the study, assessed for inclusion and exclusion criteria, and obtained written informed consent. After enrollment, and prior to initiation of the intervention, participants completed baseline self-report measures of clinical outcomes (see below). Participants then completed a total of seven PP exercises and seven phone sessions over the 12 weeks, as described previously.

#### 2.5.4. Study Outcome Assessments

Feasibility and acceptability (the main aims of this proof-of-concept study) were assessed by examining rates of exercise completion among participants as recorded by study interventionists. In addition, participants rated their optimism and positive affect immediately prior to and after each exercise on a 1–10 Likert scale. Following the exercise, they also rate the ease and overall utility of the exercise on a 1–10 scale. At week 12, open-ended feedback about the overall intervention's ease, utility, and applicability to T2D was also elicited from participants.

In addition, as a secondary aim, we explored pre-postchange in patient-reported clinical outcomes. At baseline, 6 weeks, and 12 weeks, noninterventionist study staff obtained the following validated measures (Table 1):

- Life Orientation Test-Revised (LOT-R [95]) to assess optimism.
- Gratitude Questionnaire-6 (GQ-6 [35]) for gratitude.
- Hospital Anxiety and Depression Scale (HADS [96]) for anxiety/depression.
- Diabetes Distress Scale (DDS [97]) for diabetes-related distress.
- Patient-Reported Outcomes Measurement Information System 10-Item Scale (PROMIS-10 [98]) for health-related quality of life and function.
- Summary of Diabetes Self-Care Activities Measure (SDSCA [99]) for diabetes self-care.
- MOS SAS (health behavior adherence) items were also repeated at 6 and 12 weeks.

#### 2.5.5. Data Analysis

All analyses will be performed following completion of all follow-up assessments.

*(1) Primary Aim*. Feasibility and acceptability (immediate impact).

*(a) Method*. Descriptive statistics (percentages, mean, median, and standard deviation) will be used to record and report rates of exercise completion and the 1–10 ratings of optimism, positive affect, ease, and utility for the individual weekly exercises (recorded weekly by interventionists). Paired *t*-tests will be utilized to assess mean pre-postchange in optimism and positive affect across exercises as a measure of acceptability and short-term impact.

*(b) Criteria*. The PP exercises will be considered feasible and well-accepted if (i) 4 of the 7 PP exercises are completed by a majority of patients and (ii) participants have a mean score of at least 6.5/10 on the rating of ease of completion across exercises. The PP exercises will be considered to have adequate impact if (i) participants' mean ratings of exercise utility are 6.5/10 and (ii) they rate optimism and positive affect significantly higher (*P* < 0.05) after exercise than before exercises, as assessed via paired *t*-test. Because the study interventionists collect these ratings (as opposed to the 6/12-week study outcome measures, which are collected by blinded staff), social desirability may influence pre/postratings. However, such ratings varied substantially depending on exercise content when this data was collected in an identical manner in past work [50].

*(c) Power Calculation*. A minimum of 60 exercises completed by 15 participants would lead to this study being powered at >96% to identify between-group differences with the moderate (Cohen's *d* = 0.5) immediate effect of PP exercise on optimism and positive affect seen in our prior PP intervention studies [50].

*(2) Secondary Aim*. Changes in clinical outcome measures.

*(a) Method*. We will compare pre-postchanges in the outcome measures at the two timepoints (6 and 12 weeks) using random effects regression models to allow for inclusion of missing data at either timepoint. We will also calculate effect sizes using Cohen's *d*.

*(b) Criteria*. We will consider the PP intervention to show promise for improving psychological well-being if mean scores on all psychological outcome measures are improved at 6 and 12 weeks compared to baseline (e.g., HADS score lower, LOT-R score higher at follow-up). This proof-of-concept study with a target recruitment of 15 participants was not designed (and was not powered) to detect statistically significant differences between baseline and later follow-up points on these outcome measures.

All statistical tests will be two-tailed and will be performed using Stata 11.2 (StataCorp, College Station, TX).

## 3. Results

**Figure 2:**
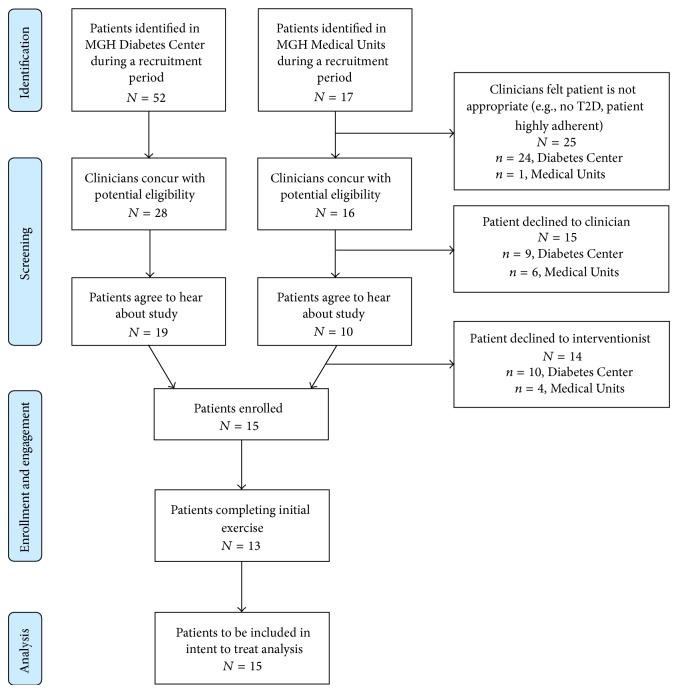
Flow diagram of enrollment.

Treatment and trainers' manuals were successfully developed for the feasibility trial, and study staff were successfully trained utilizing the above protocol.  Figure 2 displays the recruitment flow for both sites. Recruitment logistics were developed initially at the outpatient site, with subsequent addition of the inpatient cardiac units once outpatient recruitment was progressing well. In total, during time periods when study staff were available for recruitment for this unfunded feasibility trial, 69 patients with T2D were identified, 44 were judged by clinicians to be potentially eligible, 29 agreed to hear about the study, and 15 were eligible and enrolled.

Table 2 displays the baseline characteristics of enrolled participants. There were no differences in the age (*t* = 0.35; *P* = 0.71), race (*χ* ^2^ = 1.86; *P* = 0.17), or gender (*χ* ^2^ = 0.47; *P* = 0.49) of participants who declined participation compared to those who enrolled; additional data on decliners was not collected.

Overall, the mean age of participants was 60.1  ±  8.8 years, 9 (60%) were women, and the majority had one or more additional cardiovascular risk factors (e.g., hypertension and hyperlipidemia). Most participants were moderately nonadherent, with a mean MOS SAS score of 11.4 out of 18, consistent with a mean rating of 3.8 out of 6 for completion of each health behavior (diet, activity, and medication adherence).

Participants had relatively low baseline optimism (LOT-R = 13.0 ± 6.8, compared to general population mean of 15.2 ± 3.8 [34]) and gratitude (GQ-6 = 35.7 ± 6.7, general population mean 36.9 ± 4.9 [35]). Participants' mean levels of depression (mean HADS-D = 7.0 ± 3.7) and anxiety (mean HADS-A = 7.9 ± 4.2) were just below the standard cutoff of 8 for clinically significant symptoms [100]. Thus far nine participants have completed the study, with six participants still undergoing study procedures; of the nine initial participants, seven (78%) fully completed at least three PP exercises. Additional data on feasibility, acceptability, and impact will be analyzed and interpreted once all participants have completed study procedures.

## 4. Discussion

### 4.1. Discussion of PP Interventions in T2D

Thus far it appears that recruitment and enrollment for a proof-of-concept study of a PP intervention in T2D is feasible in both inpatient and outpatient settings, and phone delivery of the intervention has been straightforward. It is not yet clear whether PP exercises will be effective in boosting positive psychological states and modifying health behaviors in patients with T2D. Controlled studies of PP interventions have led to increased positive psychological well-being in a wide variety of populations [41], and we found substantial changes in positive affect over the course of 8 weeks in a pair of PP intervention studies completed in patients with cardiovascular disease [42, 101]. Furthermore, a small randomized trial (*N* = 43) of an online positive affect skills intervention led to improvements in depressive symptoms in T2D patients [54]. These data suggest that PP interventions may be able to modify positive psychological well-being, even in patients with medical illness. Even more promising are the findings from randomized trials [47, 48] of PP-type interventions in patients with cardiovascular illness that found such interventions to be associated with significantly greater improvements in health behaviors (e.g., physical activity [48] and medication adherence [47]) than in the control conditions. Empirical studies are still needed to determine whether these promising interventions are effective in improving T2D-related health behaviors.

### 4.2. Comparison of PP Interventions with Related Interventions

PP interventions may overlap to some degree with other commonly used psychological interventions. One such intervention is mindfulness-based stress reduction (MBSR), which has been successful in improving symptoms in a variety of medical conditions [102–105]. MBSR focuses on several mindfulness techniques and relaxation exercises in an intensive intervention format typically requiring eight weekly 1.5-hour sessions and a commitment to daily homework completion [106]. MBSR also typically requires extensive provider training programs that include practicums and retreats [106].

PP is distinct in important ways. In contrast to MBSR, PP exercises are briefer and can be completed independently, and in-person sessions are not required. The provider training process (completed via a training manual, role play, and audiotaped practice sessions for PP) is much simpler than that required for MBSR, improving applicability to real-world settings. Finally, as opposed to a focus on mindfulness, PP specifically targets behaviors (e.g., leveraging past success) and positive attributes (e.g., positive affect and optimism) that have been associated with increased participation in health behaviors and superior medical outcomes [26, 28, 29]. Social cognitive theory interventions that focus on self-efficacy, outcome expectations, barriers, and goal setting are also similar to PP [107, 108]. However, PP interventions have a focus on validated exercises that target additional specific positive constructs beyond optimism or self-efficacy (e.g., positive affect) linked to beneficial outcomes [108, 109]. Still, there have not been comparative effectiveness trials of the wide array of psychological interventions that aim to increase self-efficacy and well-being, and caution is warranted before clear distinctions can be made in terms of feasibility and efficacy.

### 4.3. MI and Related Health Behavior Interventions

MI is a patient-centered method for enhancing intrinsic motivation to change and facilitating behavior change [110]. MI has several features that distinguish it from other interventions. First, it is a patient-centered approach, specifically focused on having patients generate reasons for change and assisting them in resolving ambivalence about changing their behavior. Direct confrontation and persuasion are discouraged in the therapist, whose role is to help the client to thoughtfully examine his or her behavior and its consequences. Motivational enhancement therapy (MET) is a brief (typically 4-session) adaptation of MI sometimes used in clinical trials that includes normative feedback to clients about their behavior. This discrepancy between normative behavior and a client's current behavior is presented and discussed in a nonconfrontational manner with the goal of engaging clients in increasing intrinsic motivation towards change [111, 112].

MI and related interventions have several appealing characteristics. First, MI can be used with patients at any stage of change, from those who feel that they currently are not motivated to increase their activity to those who are highly motivated to increase activity but have failed on prior attempts [113]. Furthermore, it has been utilized for many years to successfully address an incredible variety of health behaviors, including physical activity, diet, and medication adherence [110]. Finally, it can be delivered remotely.

All of these factors make MI attractive to T2D patients who are poorly adherent to key health behaviors. By being adaptable to patients at any stage of change, it can apply to a large number of patients. Furthermore, as with PP, the ability to deliver MI remotely is very well suited to T2D patients, who may have functional, time, financial, or transportation barriers that preclude frequent inpatient visits.

MI has been successfully used to target physical activity and other health behaviors in T2D patients in several studies [114], but MI alone may not always be sufficient to effect change. While MI has been associated with improvements, the effect size of this technique has only been moderate (e.g., ES for physical activity = 0.19) [115]. These moderate effects may not be enough to result in changes in major downstream outcomes (e.g., reduced admissions or mortality). Furthermore, MI is not effective for all patients. MI studies have been mixed, with some studies finding no greater effect of MI compared to control conditions [116, 117]. There appear to be specific characteristics of patients who may struggle to benefit from health behavior interventions like MI, such as those who have low expectation of improvement and low overall optimism and those who have low perceived social support [118–120].

Fortunately, MI and MET have been successfully combined with additional interventions in numerous populations, including interventions targeting weight control or health behaviors in those with medical illness. For example, in a comparative effectiveness trial, MET alone was not effective in improving outcomes in patients with type 1 diabetes, but when combined with a second intervention it did result in improved clinical outcomes [121]. Additional approaches that may directly affect health behaviors and address risk factors for MI and MET nonresponse are warranted.

### 4.4. Future Directions: Combining PP and MI Interventions for Behavior Change in T2D

A novel health behavior intervention that combines MI and PP in T2D may be very effective. Physical activity may be an ideal target for such an intervention, given that positive states may be most linked with physical activity among the health behaviors [122, 123] and given the substantial evidence base for MI on physical activity. This combined PP-MI intervention could be delivered by phone, maintaining broad accessibility. If this practical intervention were to be well accepted and increase physical activity among T2D patients, this could substantially reduce rates of complications and adverse events in a high-risk population.

**Figure 3:**
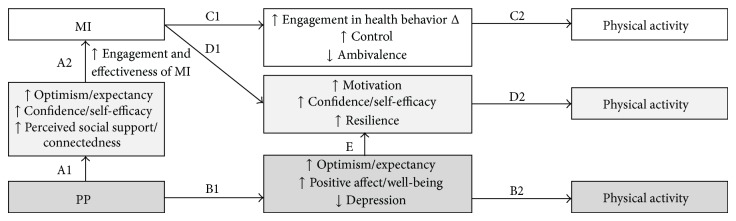
Conceptual model for the combination of positive psychology and motivational interviewing to improve physical activity.

Figure 3 outlines a model by which a combined PP-MI intervention may cultivate physical activity. First, PP interventions could boost the efficacy of standard MI. PP exercises have led to increased optimism and outcome expectancy [124, 125], confidence and self-efficacy [126], and greater perceived social support and interpersonal connectedness (pathway A1) [127, 128]. These individual and social-level constructs, in turn, have been associated with greater efficacy and impact of MI and other health behavior interventions (pathway A2) [118–120, 129, 130]. Regarding direct effects of PP on activity, the established benefits of PP on mood, resilience, well-being, and optimism (pathway B1) [41, 54, 124, 125] are associated with increased physical activity (pathway B2) [48, 88, 123, 131, 132].

Furthermore, MI itself leads to decreased ambivalence, greater intention to change, and increased feelings of control (pathway C1) [113, 133–137], all of which are consistently associated with greater physical activity (pathway C2) [138–140]. In addition, both MI (pathway D1) [113, 133, 141–145] and the previously described effects of PP on mood, well-being, and optimism (pathway E) [52, 95, 146] are associated with greater self-efficacy, confidence, and motivation, which are in turn linked to physical activity (pathway D2) [139, 147–151].

This combination of a psychological component (that can boost optimism, confidence, and positive experiences) alongside a more tightly focused, goal-oriented, and cognitive MI component may be much more powerful in this complex and vulnerable population than either approach alone. Prior attempts to combine health behavior interventions with psychological interventions have been successful in T2D but thus far have been designed only for minority of patients with clinical depression [152]. PP and MI have never been combined and evaluated in a T2D population.

### 4.5. Limitations of PP Interventions

PP interventions, alone or combined with other behavioral interventions, have several limitations. Not all patients may be interested in activities to boost their affect and well-being, and, despite the exercises' straightforward nature, not all patients may be willing or able to complete positive activities on a regular basis. Furthermore, though there is ample data linking positive psychological states to health behaviors, there is less evidence regarding the impact of PP interventions on physiologic outcomes (e.g., inflammation) important to health. Overall, this line of work is still in very early stages and PP interventions (alone or combined) are far from established health-promoting interventions. Limitations of our specific proof-of-concept trial also include the use of only a single inpatient and a single outpatient site in an academic medical center and the fact that the majority of participants were white; these factors may limit generalizability of findings.

## 5. Conclusions and Next Steps

In sum, there is evidence to suggest that higher levels of positive affect, optimism, and well-being can lead to improved health behavior adherence (and outcomes) in patients with chronic illnesses like T2D. In addition, PP interventions appear to reliably increase these positive states, have been well accepted in other populations, and are distinct from other behavioral interventions in their ease of administration, high acceptability, and specific focus on promoting positive psychological well-being.

**Figure 4:**
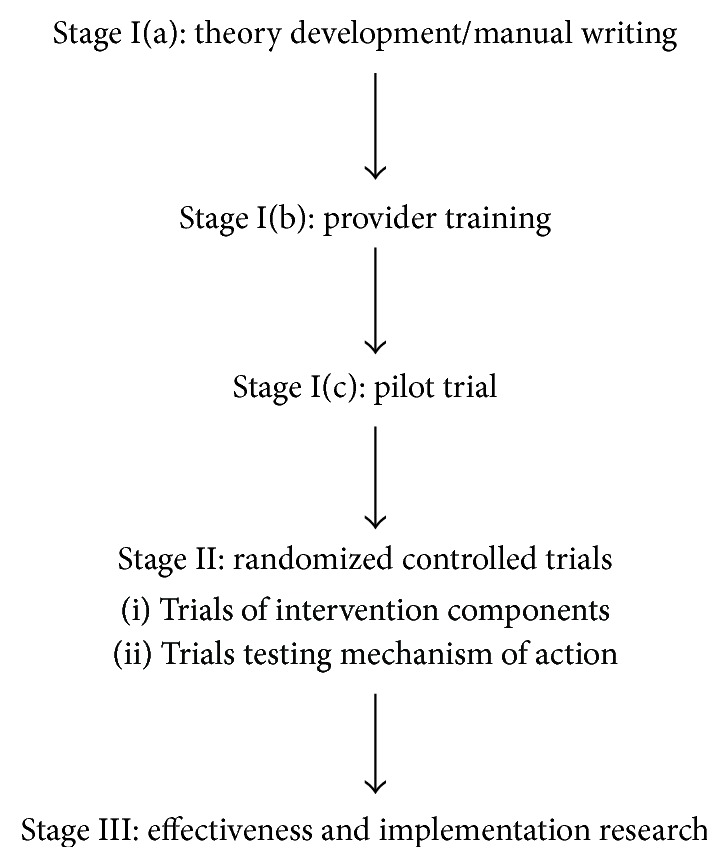
Adapted Rounsaville model of behavioral intervention development.* Note*. Adapted from [153].

However, we have just begun to test PP interventions in patients with T2D and suboptimal health behavior adherence, and it is likely that additional customization of the intervention more specifically to T2D will be useful. Utilizing the Rounsaville model [153] of behavioral intervention development (Figure 4), next steps will be to further refine and adapt the intervention and to explore its effects on health behavior when compared against a control condition among T2D patients. If the PP intervention shows promise in an initial randomized trial, larger studies focused on major clinical outcomes and clinical implementation of such programs may be warranted. As described, another potential use is that PP could be combined with other behavioral interventions (e.g., MI) to boost engagement and adherence with little added burden. Additional research is needed to identify the optimal use and dose of these interventions for patients living with T2D.

## Figures and Tables

**Box 1:**
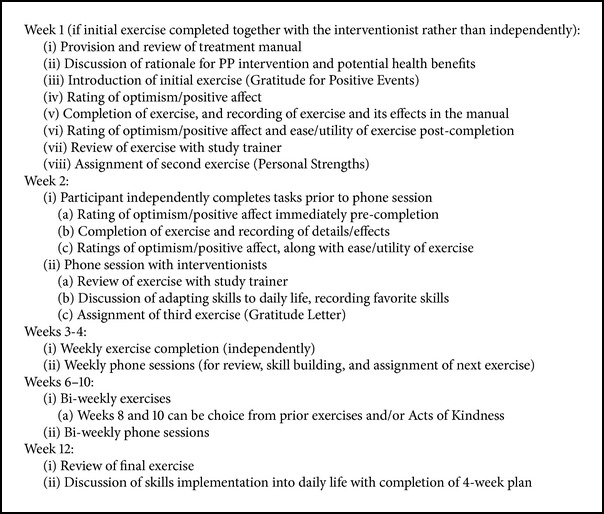
**Box 1: **Positive psychology intervention outline.

**Box 2:**
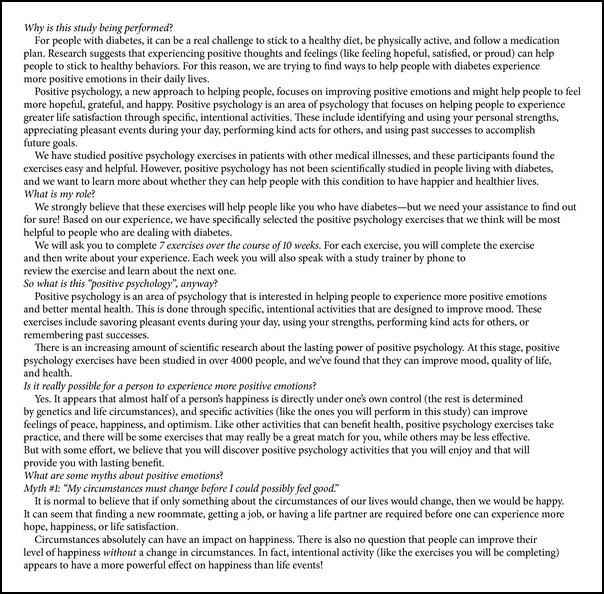
**Box 2: **Sample introductory page from the PP manual.

**Box 3:**
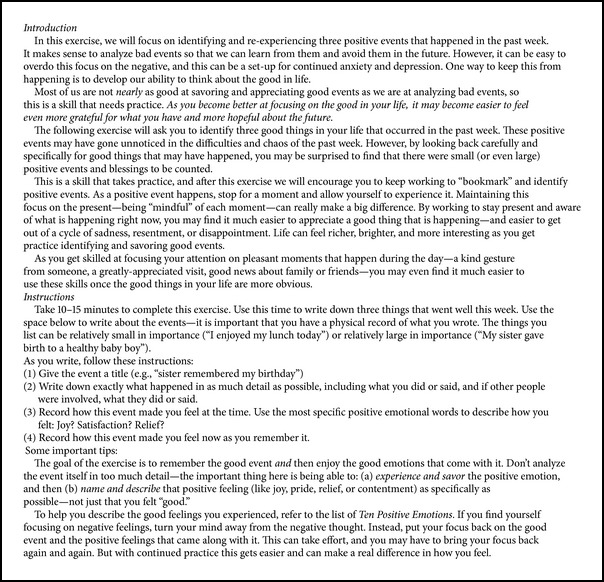
**Box 3: **Sample exercise rationale and instructions.

**Table 1:** Schedule of study events.

Event	Week								
	Preenrollment	1	2	3	4	6	8	10	12
Adherence assessment (MOS SAS)	X					X			X

Cognitive screening	X								

Chart review Baseline characteristics		X							

PP exercise Completed independently		X	X	X	X	X	X	X	∗

PP exercise ratings Completed before/after exercise		X	X	X	X	X	X	X	∗

Psychological self-report measures (HADS, LOT-R, and GQ-6)		X				X			X

Medical self-report measures (PROMIS-10, SDSCA, and DDS)		X				X			X

^∗^Week 12 involves review of the final (week 10) exercise and exercise ratings.

*Note*. GQ-6: Gratitude Questionnaire-6; HADS: Hospital Anxiety and Depression Scale; DDS: Diabetes Distress Scale; LOT-R: Life Orientation Test-Revised; MOS SAS: Medical Outcomes Study Specific Adherence Scale items. PROMIS-10: Patient-Reported Outcomes Measurement Information System 10-Item Scale; SDSCA: Summary of Diabetes Self-Care Activities Measure.

**Table 2:** Baseline sociodemographic and clinical characteristics.

Characteristics	*N* (%)^∗^ (total *N* = 15)
Demographics and psychosocial characteristics	
Age in years (mean (SD))	60.1 (8.8)
Male	6 (40)
White	14 (93.3)
Medical history	
Hypertension	14 (93.3)
Hyperlipidemia	10 (66.7)
Coronary artery disease	3 (20)
Current smoking	1 (6.7)
Body mass index	31.03 (5.5)
Hemoglobin A1c	8.7 (1.6)
Medications	
Aspirin	11 (73.3)
ACE inhibitor/angiotensin II receptor blocker	10 (66.7)
Lipid-lowering agent (e.g., statin)	13 (86.7)
Insulin	12 (80)
Oral hypoglycemic agents	9 (60)
Antidepressants	6 (40)
Baseline psychological self-report measures (mean (SD))	
LOT-R (range 0–24, higher = greater optimism)	13.0 (6.8)
GQ-6 (range 6–42, higher = greater gratitude)	35.7 (6.7)
HADS-D (range 0–21, higher = more depression)	7.0 (3.7)
HADS-A (range 0–21, higher = more anxiety)	7.9 (4.2)
Baseline medical self-report measures (mean (SD))	
MOS SAS (range 3–18, higher = more adherent)	11.4 (3.4)
PROMIS-10 subscales (range 4–20, higher = better health)	
Global Physical Health	11.8 (3.2)
Global Mental Health	10.7 (1.9)
DDS subscales (range 1–6, higher = more distress)	
Emotional	3.1 (1.4)
Physical	1.1 (0.1)
Regimen	3.0 (1.4)
Interpersonal	2.4 (1.7)
SDSCA subscales (range 0–7, higher = better adherence)	
Diet	4.1 (1.8)
Exercise	1.3 (1.9)
Blood sugar	5.2 (2.4)
Foot care	2.7 (2.8)

^∗^All figures are *N* (%) unless otherwise specified.

*Note*. ACE: angiotensin-converting enzyme; DDS: Diabetes Distress Scale; GQ-6: Gratitude Questionnaire-6; HADS-A: Hospital Anxiety and Depression Scale-Anxiety Subscale; HADS-D: Hospital Anxiety and Depression Scale-Depression Subscale; LOT-R: Life Orientation Test-Revised; MOS SAS: Medical Outcomes Study Specific Adherence Scale items; PROMIS-10: Patient-Reported Outcomes Measurement Information System 10-Item Scale; SDSCA: Summary of Diabetes Self-Care Activities.
